# *FTO* Is Associated with Aortic Valve Stenosis in a Gender Specific Manner of Heterozygote Advantage: A Population-Based Case-Control Study

**DOI:** 10.1371/journal.pone.0139419

**Published:** 2015-10-02

**Authors:** Cindy Thron, Payam Akhyari, Erhard Godehardt, Artur Lichtenberg, Ulrich Rüther, Stefanie Seehaus

**Affiliations:** 1 Institute for Animal Developmental and Molecular Biology, Heinrich-Heine-University, Düsseldorf, Germany; 2 Department of Cardiovascular Surgery, Heinrich-Heine-University, Düsseldorf, Germany; Ohio State University Medical Center, UNITED STATES

## Abstract

**Background:**

Single nucleotide polymorphisms (SNPs) within the *Fat mass and obesity associated* (*FTO*) gene have been linked with increased body weight. However, the data on an association of *FTO* with cardiovascular diseases remains conflicting. Therefore, we ascertained whether *FTO* is associated with aortic valve stenosis (AVS), one of the most frequent cardiovascular diseases in the Western world.

**Methods and Findings:**

In this population-based case-control study the *FTO* SNP rs9939609 was analyzed in 300 German patients with AVS and 429 German controls of the KORA survey S4, representing a random population. Blood samples were collected prior to aortic valve replacement in AVS cases and *FTO* rs9939609 was genotyped via ARMS-PCR. Genotype frequencies differed significantly between AVS cases and KORA controls (*p* = 0.004). Separate gender-analyses uncovered an association of *FTO* with AVS exclusively in males; homozygote carriers for the risk-allele (A) had a higher risk to develop AVS (*p* = 0.017, odds ratio (OR) 1.727; 95% confidence interval (CI) 1.087–2.747, recessive model), whereas heterozygote carriers for the risk-allele showed a lower risk (*p* = 0.002, OR 0.565, 95% CI 0.384–0.828, overdominant model). After adjustment for multiple co-variables, the odds ratios of heterozygotes remained significant for an association with AVS (*p* = 0.008, OR 0.565, 95% CI 0.369–0.861).

**Conclusions:**

This study revealed an association of *FTO* rs9939609 with AVS. Furthermore, this association was restricted to men, with heterozygotes having a significantly lower chance to develop AVS. Lastly, the association between *FTO* and AVS was independent of BMI and other variables such as diabetes mellitus.

## Introduction

Interrelated multifactorial diseases like obesity, cardiovascular diseases, metabolic syndrome, type 2 diabetes and cancer have emerged as major diseases globally with severe effects on health care costs. In order to better understand these diseases, genome-wide association studies were started to unravel these complex diseases by identifying candidate genes. In obesity, common variants in the *Fat mass and obesity associated* (*FTO*) gene were found to have the highest effect on body weight [1–3]. Homozygote risk-allele carriers (AA) of the *FTO* single nucleotide polymorphism (SNP) rs9939609 showed a 1.67-fold increased risk to develop obesity in comparison to non-risk-allele carriers (TT) [2] which could be reproduced for several *FTO* SNPs in different ethnic populations [4–6]. Additionally, murine loss and gain of function analyses confirmed FTO’s role in body weight regulation [7, 8]. Nevertheless, FTO’s cellular function as a *N6*-methyladenosine RNA demethylase [9] remains poorly understood. Despite its ubiquitous expression in different organs with the greatest expression in the hypothalamus, an area important for controlling energy metabolism and food intake [2, 10, 11], data on FTO expression in relation to experimentally altered nutritional states have been conflicting [10, 12, 13].

Since most GWAS can only detect a locus but not a gene the question arose whether *FTO* is “the sole” obesity gene. A potential candidate in close 5’ proximity to *FTO* would be *RPGRIP1L*. Initial studies identified a transcription factor, possibly regulating *FTO* and *RPGRIP1L* expression by binding to SNPs within *FTO* [14, 15]. However, several other studies could not confirm an association of *FTO* SNPs with expression of *RPGRIP1L* [16, 17] or altered *Rpgrip1l* expression in *Fto*-deficient mice [8]. An interaction of the obesity-associated intronic region of *FTO* and the promoter sequence of *Iroqouis 3* (*IRX3*) has recently been identified [18]. Furthermore, *FTO* SNPs could be associated with expression of *IRX3* in human brains, while *Irx3*-deficient mice showed a phenotype similar to *Fto*-deficient mice. These results suggest that *IRX3* could be a functional long-range target of *FTO* SNPs. However, analyses in human brain tissues have been restricted to the cerebellum and have not focused on the hypothalamus. Thus, further studies are necessary to answer the question if either *FTO* or *IRX3*, or both are “the” obesity gene(s).

Obesity is a major risk factor for the development of cardiovascular diseases. For this very reason the question arose whether *FTO* genotype might bear a direct risk for cardiovascular diseases. First data on this have been inconsistent: showing either a BMI-independent [19, 20] or a BMI-dependent [21, 22] association of *FTO* with cardiovascular diseases. Furthermore, only few studies referred to a specific cardiovascular disease.

Among cardiovascular diseases aortic valve stenosis (AVS) is one of the most prominent in the Western world [23]. The probability of developing AVS increases with age and male gender [24]. In 2013 one third of German cardiac surgical procedures were on heart valves with 72% due to AVS alone [25]. Severity increases with symptomatic AVS leading to a death rate of more than 50% when immediate aortic valve replacement is not performed [26]. There are no medical therapies known to slow down or avert the progression of AVS with molecular mechanisms being poorly understood. Although familial clustering of individuals has been reported [27], little is known about genetic factors contributing to the development of AVS.

It remains unclear whether increased BMI leads to an association of *FTO* with cardiovascular diseases or if *FTO* has a direct impact. Because AVS is one of the most prominent cardiovascular diseases the aim of this study was to determine a possible association of *FTO* with AVS. A better understanding of how *FTO* and *IRX3* impacts AVS is a first step towards a better treatment of AVS in the future.

## Methods

### Study Populations

All participants, AVS cases and KORA controls, of this prospective study were of Western European descent and resided in Germany. Prior to this study, a pilot study was carried out to determine the appropriate number of cases and controls (S1 File). The study plan was approved by the ethics committee of the Medical Faculty of the Heinrich-Heine-University (study number 3428) following the guidelines of the Helsinki Declaration.

During the study period 105 females and 195 males diagnosed with AVS who underwent heart valve replacement at the Department of Cardiovascular Surgery at the University Hospital Düsseldorf gave their written informed consent to participate in this study. For enrollment of patients the primary inclusion criteria was diagnosed AVS. This diagnosis was based on a triple-secured diagnostic chain: 1) manual chart review and review of medical records of all pre-admission records, including pre-operative transthoracic echocardiography (TTE), transesophageal echocardiography (TEE) or left heart catheterization; 2) in-house TTE and intraoperative TEE; 3) intraoperative macroscopic assessment of the aortic valve by the involved senior surgeon. Details of the prospective process for identification of patients with AVS are described in (S1 File). Criteria for exclusion were patients younger than 18, confirmed infection with HIV or hepatitis, previous surgery on one or more heart valves, mental health disease, drug addiction, pregnancy and breast feeding.

The control group represents a random population sample provided by the population-based health survey “KORA-gen” at the Helmholtz Zentrum München (Germany). KORA control definition and study design have been described previously [28, 29]. In brief, data of 600,000 participants were collected in four surveys over 17 years, which included standardized interviews, medical and laboratory examinations and biosamples. In these studies, several cardiovascular diseases, including cardiac infarction, stroke, angina pectoris, PAD and heart failure have been examined. In the most recent KORA survey (S4), 2,200 healthy participants were selected to serve as a control pool for genetic analyses. Several clinical studies were published using this control population thus far. Out of these studies, 129 female and 300 male KORA controls with a median age of 65 years were chosen for this study.

Acquisition of clinical variables is described in detail within (S1 File).

### Genotyping

Blood samples were taken from AVS cases before heart valve replacement. DNA was purified from whole blood samples using a Qiagen blood kit (Gentra Puregene Blood Kit, # 158467, Qiagen, Hilden, Germany) according to manufacturer’s protocol. *FTO* rs9939609 was genotyped via tetra-primer amplification refractory mutation system polymerase chain reaction (ARMS-PCR) as described earlier [30], (S1 File and S1 Table). *FTO* rs8050136 and rs17817449 served as internal genotyping controls being part of the same linkage disequilibrium block as rs9939609 [1, 2]. Genotypes of KORA controls were supplied by KORA-gen.

### Statistical Analyses

Demographic and clinical data of AVS cases and KORA controls were tested for normal distribution with a Shapiro-Wilk test and for homogeneity of variance with a Levene’s test. Values are given as mean ± standard deviation (SD). AVS cases were compared to KORA controls with standard statistical testing methods (Student’s t-Test, Mann-Whitney-U Test and Fisher’s-Exact-Test).

Genotype frequency of *FTO* rs9939609 was tested for deviation from Hardy-Weinberg equilibrium (HWE) using the χ^2^ test to exclude possible error rates of SNP genotyping in KORA controls [31–33] and to identify a possible gene-disease association in AVS cases [34–36]. The null hypothesis was tested with an overall test (co-dominant model) by applying the Fisher’s-Exact-Test. Based on the results, the recessive and overdominant model were further analyzed. The additive model was not analyzed further due to the lack of a linear trend of the risk-allele when applying the co-dominant model initially. Deviation of genotype frequency between AVS cases and KORA controls was corrected for age, BMI, diabetes mellitus and hypertension in logistic regression models. The strength of association was calculated with odds ratio (OR) and 95% confidence interval (CI), adjusting for the multiple co-variables. The value of each co-variable of each individual has been used for all logistic regression models. Blood lipid levels have not been included in logistic regression models, since levels in AVS cases were lower.

Statistical analyses were conducted using SPSS (version 21.0.0.1 SPSS Inc., Chicago, IL, USA) and R (version 3.1.1, www.r-project.org). Power calculations are further described within (S1 File).

## Results

### Baseline Data of AVS Cases and KORA Controls

Demographic and clinical data of AVS cases and KORA controls are summarized in Table 1. As expected AVS cases were older with increased prevalence for diabetes mellitus and hypertension (all were *p*<0.001). Nevertheless, AVS cases had significantly lower BMI and blood lipid levels (BMI and cholesterol levels *p*<0.001, triglycerides *p* = 0.005). Similar results were obtained by others when comparing baseline data of patients with acute coronary syndrome and controls representing a random population in this study [19]. Appearance of peripheral artery disease and smoking status did not differ between AVS cases and KORA controls (*p* = 0.511 and *p* = 0.381 respectively).

**Table 1:** Demographic and Clinical Data of AVS Cases and KORA Controls.

Variables	AVS cases	KORA controls	*p*-value
Male/female [n]	195/105	300/129	0.171^1^
(n)	(300)	(429)	
Age [years]	71.3±10.1	64.9±3.2	<0.001^2^
(n)	(300)	(429)	
BMI [kg/m^2^]	27.1±4.1	28.8±4.0	<0.001^2^
(n)	(297)	(429)	
Cholesterol [mmol/l]	4.0±1.1	6.2±1.1	<0.001^3^
(n)	(282)	(425)	
LDL [mmol/l]	2.4±0.9	3.9±1.0	<0.001^3^
(n)	(274)	(425)	
HDL [mmol/l]	1.1±0.4	1.4±0.4	<0.001^2^
(n)	(274)	(425)	
Triglycerides [mmol/l]	1.4±0.7	1.6±1.0	0.005
(n)	(282)	(418)	
Diabetes mellitus n [%]	80 [26.8]	30 [7.0]	<0.001^1^
(n)	(299)	(427)	
Hypertension n [%]	222 [74.7]	205 [48.4]	<0.001^1^
(n)	(297)	(425)	
PAD n [%]	30 [10.1]	35 [8.5]	0.511^1^
(n)	(297)	(410)	
Smoking n [%]	32 [10.8]	47 [11.0]	0.381^1^
Smoked formerly n [%]	103 [34.7]	196 [45.8]	
(n)	(297)	(428)	

Two-sided *p*-values were calculated using

^1^Fisher’s-Exact-Test

^2^Mann-Whitney-U Test and

^3^Student’s t-Test. Values are mean±SD. PAD, peripheral artery disease.

### *FTO* rs9939609 Genotype Frequencies of AVS Cases and KORA Controls

In the KORA controls, the genotypic frequency of *FTO* rs9939609 did not deviate from HWE (*p* = 0.277, S2 Table), whereas a significant deviation was found in the AVS cases (*p* = 0.001).

The accuracy of genotyping was confirmed with two additional SNPs in linkage disequilibrium with *FTO* rs9939609 (S3 Table). The genotypic distribution of *FTO* rs9939609 differed significantly between AVS cases and KORA controls (*p* = 0.004; OR 0.614, 95% CI 0.433–0.868 for TA genotype; OR 1.042, 95% CI 0.672–1.614 for AA genotype; Table 2 and S4 Table). AVS cases showed a higher proportion of the high-risk genotype (AA) compared to KORA controls (21.7% versus 16.8%, Table 2). However, this difference was not significant in the recessive model (*p* = 0.102, Table 2). The TA genotype was associated with a lower risk for developing AVS and significantly fewer TA individuals were found in the AVS population than in the KORA control population in the overdominant model (39.3% versus 51.7%, *p* = 0.001, OR 0.605, 95% CI 0.443–0.824, Table 2 and S4 Table). In a logistic regression controlling for age, BMI, diabetes mellitus and hypertension the association of TA with lower risk of developing AVS remained (*p* = 0.030, OR 0.675, 95% CI 0.472–0.962, Table 2 and S4 Table), whereas significance in the co-dominant model was lost (*p* = 0.092, Table 2).

**Table 2:** *FTO* rs9939609 Genotype Frequencies in AVS Cases and KORA Controls.

Genotype distribution AVS cases n [%]							Unadjusted^1^/adjusted^2^		
KORA controls n [%]							*p*-value of genetic model		
Gender	TT		TA		AA		Co-dominant	Recessive	Overdominant
All	117	[39.0]	118	[39.3]	65	[21.7]	0.004/0.092	0.102/0.224	0.001/0.030
(n = 729)	135	[31.5]	222	[51.7]	72	[16.8]			
Male	70	[35.9]	74	[37.9]	51	[26.2]	0.005/0.030	0.017/0.157	0.002/0.008
(n = 495)	93	[31.0]	156	[52.0]	51	[17.0]			
Female	47	[44.8]	44	[41.9]	14	[13.3]	0.170/0.940	0.584/0.964	0.188/0.766
(n = 234)	42	[32.6]	66	[51.2]	21	[16.3]			

^1^Two-sided *p*-values were calculated with the Fisher's-Exact-Test.

^2^By using logistic regression models *p*-values were adjusted for age, BMI, diabetes mellitus and hypertension.

### *FTO* rs9939609 Genotype Frequencies of Male and Female AVS Cases and KORA Controls

*FTO* rs9939609 genotype frequencies of males and females were analyzed separately due to known gender differences for developing cardiovascular diseases, especially for AVS. A significant deviation from HWE in genotypic frequencies of *FTO* rs9939609 persisted in male AVS cases, but was not observed in female AVS cases (*p* = 0.001 and *p* = 0.516 respectively, S2 Table).

The genotypic frequency of *FTO* rs9939609 differed significantly in males (*p* = 0.005; OR 0.631, 95% CI 0.407–0.976 for TA genotype; OR 1.327, 95% CI 0.784–2.250 for AA genotype; not adjusted for age, BMI, diabetes mellitus and hypertension; Tables 2 and 3) likewise after adjustment for these factors (*p* = 0.030; OR 0.578, 95% CI 0.357–0.930 for TA genotype; OR 1.058, 95% CI 0.598–1.867 for AA genotype), but not in females (*p* = 0.170, Table 2). The percentage of the AA genotype in male AVS cases was elevated compared to KORA controls in the recessive model (26.2% versus 17.0%, *p* = 0.017, OR 1.727, 95% CI 1.087–2.747, Tables 2 and 3). However, results were non-significant after adjustment for co-variables (*p* = 0.157, OR 1.438, 95% CI 0.867–2.379). Evidence of heterozygote advantage persisted in male AVS cases in the adjusted overdominant model (37.9% versus 52.0%, *p* = 0.008, OR 0.565, 95% CI 0.369–0.861).

**Table 3:** Odds Ratios (OR) and 95% Confidence Intervals (CI) of *FTO* rs9939609 Between Male AVS Cases and KORA Controls.

		Unadjusted		Adjusted^1^	
Genetic model	Genotype	OR	[95% CI]	OR	[95% CI]
Co-dominant	TT	1		1	
	TA	0.631	[0.407–0.976]	0.578	[0.357–0.930]
	AA	1.327	[0.784–2.250]	1.058	[0.598–1.867]
Recessive	TT+TA	1		1	
	AA	1.727	[1.087–2.747]	1.438	[0.867–2.379]
Overdominant	TT+AA	1		1	
	TA	0.565	[0.384–0.828]	0.565	[0.369–0.861]

^1^Values were adjusted for age, BMI, diabetes mellitus and hypertension.

### *FTO* rs9939609 Genotype Frequencies of 50 to 70-Year-Old Men

AVS is a cardiovascular disease that increases with age. Therefore it is not surprising that AVS cases of this study showed a higher mean age than KORA controls (71.2 years versus 64.9 years, Table 1). To exclude the effect of age on AVS *FTO* rs9939609 genotypic frequencies of male AVS cases and KORA controls, individuals between 50–70 years were analyzed separately.

In these analyses the AA genotype was significantly associated with higher risk to develop AVS in the unadjusted recessive model (*p* = 0.002, OR 2.059, 95% CI 1.090–3.820, S5 Table), but was not significant after adjustment for co-variables (*p* = 0.062, OR 1.839, 95% CI 0.958–3.463). Nevertheless, the presence of heterozygote advantage persisted in male AVS cases after adjustment for co-variables (*p* = 0.002, OR 0.395, 95% CI 0.216–0.702).

## Discussion

With this case-control study of participants residing in Germany, we demonstrate for the first time an association between *FTO* rs9939609 and AVS, exclusively restricted to men. Strong association of *FTO* with AVS was found in the overdominant model, with lower risk of developing AVS in TA heterozygotes. This effect was significantly higher than the association in AA carriers independent of age, BMI, diabetes mellitus and hypertension.

With three different approaches we minimized possibilities for false positive and negative results of an association of *FTO* with AVS. First, KORA controls were in HWE. Second, the power of the study was sufficient. Third, genotyping mistakes have been excluded via genotyping with two additional SNPs in linkage disequilibrium with *FTO* rs9939609, namely rs8050136 and rs17817449 [1, 2]. Furthermore, multiple association studies used the test of HWE to exclude possible error rates of SNP genotyping for the cases. In contrast Lee proposed screening the genome for disease-susceptibility genes by testing deviation from HWE in a gene bank of any disease [37] as supported by others [34–36]. Therefore we argue that association of *FTO* with AVS is evident by significant deviation from HWE in AVS cases.

Of all AVS study participants, 65% were male, which reflects the gender-specific proportions observed in the largest German national database for surgical and interventional procedures on the aortic valve and underlines the higher risk of AVS for men in comparison to women [38]. Surprisingly, the association of *FTO* with AVS was restricted to men. Such gender differences in *FTO* association studies have been reported previously. For example, variation at the SNP rs9939609 was associated with BMI in females, but not males, affecting children, adolescents and adults of different ethnic origin [39–42]. A study on *FTO* rs17817449 revealed an interaction between SNP and physical activity on BMI in males but not females [43]. Differences in hormone levels, fat mass storage or physical activity could contribute to the differences in association studies for *FTO*. Due to these results we propose to identify gender differences by first analyzing both genders together and then separately in association studies for *FTO*. With this approach missing a significant effect of *FTO* due to analyses of non-affected gender by chance could be avoided. These considerations could further explain previous conflicting results on an association of *FTO* with cardiovascular diseases in which separate gender analyses were intentionally not performed [19, 21, 44]. Nevertheless, we were able to reveal a gender specific role of *FTO* in the context of therapeutically relevant AVS.

An association of *FTO* with AVS resulted in a reduced risk for developing AVS in men assuming the overdominant model. To our knowledge, this finding has not been discussed in any other genetic association study of *FTO* with cardiovascular diseases. The most prominent example of heterozygote advantage is a protection from severe symptoms of malaria in heterozygote carriers of sickle-cell anemia whereas carriers of the homozygote risk-allele suffer from severe symptoms due to sickle-cell disease [45]. So far, three other *FTO* association studies have found an effect of heterosis. One study analyzed the effect of *FTO* genotype with fat-related traits in an experimental pig population [46]. Fat weight, fat depth and fat area of different fat storages were associated with the SNP in heterozygotes. In a second study, rs9939609 was associated with BMI in European girls but not boys, whereas heterozygote boys profited from lower BMI and other obesity-related traits [39]. The third study identified an association of rs9939609 with cardiovascular diseases in males [20]. Taking a closer look at the results reveals a positive effect of heterosis with higher significance in different adjusted models than the association of the homozygote risk-allele genotype, although this was not discussed in the publication. The importance of the specific type of cardiovascular disease analyzed for an association with *FTO* should not be underestimated. According to the study [20], it was unclear whether study participants suffered from cardiovascular diseases similar in their etiology to AVS or whether they specifically suffered from AVS. Moreover, the proportion of the respective subgroups were not reported.

Whether TA carriers are at lower risk for AVS due to a broader range of *FTO* expression in different cells or tissues in comparison to homozygotes remains unclear. One possible explanation could be a deleterious effect of too little or too much *FTO* expression within the heart or aortic valve leaflets. So far, differences of *FTO* expression, dependent on the risk-allele genotype, were found in unspliced nuclear RNA of human fibroblasts [16]. In these studies, *FTO* expression for the risk-allele was elevated compared to the non-risk-allele; whether *FTO* SNPs are directly connected to *FTO* expression and if this is the case in aortic valve leaflets remains unknown. A third explanation for heterozygote advantage could be even the involvement of an additional factor [47, 48] or an interaction of the *IRX3* promoter with the obesity-associated SNP region of *FTO* [18]. In this study, array expression data revealed that *IRX3* was highly expressed within the heart at levels approximately 8-fold higher than the median expression across several human tissues. In comparison *IRX3* expression within the cerebellum was approximately 50% lower than the median expression. Thus, a role of *IRX3* in altering the development of AVS should be considered for future molecular analyses. In terms of function, IRX3 has been shown to be a direct repressor of transcription of *Connexin 43* (*CX43*) in human hearts [49]. Based on these findings, an impact of *FTO* SNPs on CX43 may be suggested which goes beyond the altered expression of CX43 found in left ventricular hypertrophy in AVS patients [50]. Furthermore, gender specific differences in expression of IRX3 and CX43 have been described [51]. Thus, we suggest that *IRX3* as a target of *FTO* SNPs may lead to altered CX43 expression, influencing progression of AVS. Nevertheless, further experiments are needed to test these predictions.

Although, rs9939609 was associated with AVS for all genetic models applied in males (especially in the subpopulation of 50–70 year old AVS cases and KORA controls), after logistic regression, the AA genotype failed to be associated with a higher risk of developing AVS. Sensitivity analyses revealed an impact of diabetes mellitus on the risk of AA carriers to develop AVS, supporting the effect of diabetes on the progression of AVS as previously suggested [52–54]. However, heterozygous males profited from a reduced risk of developing AVS, despite the considerable number of other risk factors identified so far. Interestingly, heterozygote advantage in European boys, uncovered by Jacobsson et al., was not only restricted to the BMI. Male individuals of the TA genotype showed decreased fasting serum insulin levels and an increased insulin sensitivity, whereas the SNP rs9939609 was associated with higher plasma glucose levels in girls [39].

With different approaches we minimized the chance for false results of an association of *FTO* with AVS. Nevertheless, an important limitation of this study is the lack of a second case cohort. Thus, false positive and negative results cannot be completely eliminated. Furthermore, we used a random control population where we cannot exclude the appearance of asymptomatic AVS in a few KORA controls due to the lack of routine echocardiography. Up to 2.8% of KORA controls could suffer from undetected AVS [55]. However, the effect of heterozygote advantage with a reduced risk to develop AVS should be even more pronounced with “true” controls. To test this and to overcome the limitation of a missing second case cohort, additional clinical studies with large cohorts of different ethnic origin who underwent routine echocardiography are needed. Further, an extended evaluation of the metabolic status, e.g. by standardized oral glucose tolerance test, due to observations in European boys [39] would more clearly delineate the disease etiology. Finally, in this study the primary inclusion criteria was the presence of AVS at the time of admission to the hospital for cardiac surgery on the aortic valve. Hence, patients with bicuspid aortic valve disease (n = 24) and patients with rheumatic valvular disease (n = 2) have been included in this study. Despite the prospective nature of this study, reliable longitudinal data are missing, making a detailed analysis with a focus on these sub-cohorts difficult.

In summary, in our case-control study we showed a gender specific association of *FTO* with AVS independent of BMI, diabetes mellitus and other co-variables. Heterozygote risk-allele carriers profit from a reduced risk to develop AVS. So far, not much is known about the genetics causing severe symptomatic AVS leading to urgent aortic valve replacement. We suggest that “a bit” of the *FTO* risk-allele is important to keep the balance of a relatively healthy aortic valve presumably through altered expression of *IRX3* within the heart. This study provides a first step towards an understanding of the genetics basis of AVS and the complexity of action of *FTO* variants on different diseases.

## Supporting Information

S1 Table Primer Sequences for Genotyping AVS Cases. (PDF)

S2 Table Hardy-Weinberg Equilibrium in Genotypic Frequencies of *FTO* rs9939609 in AVS Cases and KORA Controls. (PDF)

S3 Table *FTO* rs9939609, rs8050136 and rs17817449 Genotype Frequencies in AVS Cases. (PDF)

S4 Table Odds Ratios (OR) and 95% Confidence Intervals (CI) of *FTO* rs9939609 Between AVS Cases and KORA Controls. (PDF)

S5 Table Odds Ratios (OR) and 95% Confidence Intervals (CI) of *FTO* rs9939609 Between 368 Male AVS Cases and KORA Controls, Age 50–70 Years. (PDF)

S1 File SI Methods. (PDF)

## References

[1] Dina C, Meyre D, Gallina S, Durand E, Korner A, Jacobson P, et al Variation in *FTO* contributes to childhood obesity and severe adult obesity. Nat Genet. 2007;39(6):724–6. 17496892 10.1038/ng2048

[2] Frayling TM, Timpson NJ, Weedon MN, Zeggini E, Freathy RM, Lindgren CM, et al A common variant in the *FTO* gene is associated with body mass index and predisposes to childhood and adult obesity. Science. 2007;316(5826):889–94. 17434869 10.1126/science.1141634 PMC2646098

[3] Scuteri A, Sanna S, Chen WM, Uda M, Albai G, Strait J, et al Genome-wide association scan shows genetic variants in the *FTO* gene are associated with obesity-related traits. PLoS Genet. 2007;3(7):e115 17658951 10.1371/journal.pgen.0030115 PMC1934391

[4] Cornes BK, Lind PA, Medland SE, Montgomery GW, Nyholt DR, Martin NG. Replication of the association of common rs9939609 variant of *FTO* with increased BMI in an Australian adult twin population but no evidence for gene by environment (G x E) interaction. Int J Obes (Lond). 2009;33(1):75–9. 19030008 10.1038/ijo.2008.223

[5] Tan JT, Dorajoo R, Seielstad M, Sim XL, Ong RT, Chia KS, et al *FTO* variants are associated with obesity in the Chinese and Malay populations in Singapore. Diabetes. 2008;57(10):2851–7. 10.2337/db08-0214 18599522 PMC2551698

[6] Villalobos-Comparan M, Teresa Flores-Dorantes M, Teresa Villarreal-Molina M, Rodriguez-Cruz M, Garcia-Ulloa AC, Robles L, et al The *FTO* gene is associated with adulthood obesity in the Mexican population. Obesity (Silver Spring). 2008;16(10):2296–301. 18719664 10.1038/oby.2008.367

[7] Church C, Moir L, McMurray F, Girard C, Banks GT, Teboul L, et al Overexpression of *Fto* leads to increased food intake and results in obesity. Nat Genet. 2010;42(12):1086–92. 10.1038/ng.713 21076408 PMC3018646

[8] Fischer J, Koch L, Emmerling C, Vierkotten J, Peters T, Brüning JC, et al Inactivation of the *Fto* gene protects from obesity. Nature. 2009;458(7240):894–8. 10.1038/nature07848 19234441

[9] Jia G, Fu Y, Zhao X, Dai Q, Zheng G, Yang Y, et al N6-methyladenosine in nuclear RNA is a major substrate of the obesity-associated FTO. Nat Chem Biol. 2011;7(12):885–7. 10.1038/nchembio.687 22002720 PMC3218240

[10] Gerken T, Girard CA, Tung YC, Webby CJ, Saudek V, Hewitson KS, et al The obesity-associated *FTO* gene encodes a 2-oxoglutarate-dependent nucleic acid demethylase. Science. 2007;318(5855):1469–72. 17991826 10.1126/science.1151710 PMC2668859

[11] Peters T, Ausmeier K, Rüther U. Cloning of *Fatso* (*Fto*), a novel gene deleted by the *Fused toes* (*Ft*) mouse mutation. Mamm Genome. 1999;10(10):983–6. 10501967 10.1007/s003359901144

[12] Fredriksson R, Hagglund M, Olszewski PK, Stephansson O, Jacobsson JA, Olszewska AM, et al The obesity gene, *FTO*, is of ancient origin, up-regulated during food deprivation and expressed in neurons of feeding-related nuclei of the brain. Endocrinology. 2008;149(5):2062–71. 10.1210/en.2007-1457 18218688

[13] Olszewski PK, Radomska KJ, Ghimire K, Klockars A, Ingman C, Olszewska AM, et al Fto immunoreactivity is widespread in the rodent brain and abundant in feeding-related sites, but the number of Fto-positive cells is not affected by changes in energy balance. Physiol Behav. 2011;103(2):248–53. 10.1016/j.physbeh.2011.01.022 21295049

[14] Stratigopoulos G, LeDuc CA, Cremona ML, Chung WK, Leibel RL. Cut-like homeobox 1 (CUX1) regulates expression of the *fat mass and obesity-associated* and *retinitis pigmentosa GTPase regulator-interacting protein-1-like* (*RPGRIP1L*) genes and coordinates leptin receptor signaling. J Biol Chem. 2011;286(3):2155–70. 10.1074/jbc.M110.188482 21037323 PMC3023512

[15] Stratigopoulos G, Padilla SL, LeDuc CA, Watson E, Hattersley AT, McCarthy MI, et al Regulation of *Fto*/*Ftm* gene expression in mice and humans. Am J Physiol Regul Integr Comp Physiol. 2008;294(4):R1185–96. 10.1152/ajpregu.00839.2007 18256137 PMC2808712

[16] Berulava T, Horsthemke B. The obesity-associated SNPs in intron 1 of the *FTO* gene affect primary transcript levels. Eur J Hum Genet. 2010;18(9):1054–6. 10.1038/ejhg.2010.71 20512162 PMC2987405

[17] Klöting N, Schleinitz D, Ruschke K, Berndt J, Fasshauer M, Tonjes A, et al Inverse relationship between obesity and *FTO* gene expression in visceral adipose tissue in humans. Diabetologia. 2008;51(4):641–7. 10.1007/s00125-008-0928-9 18251005

[18] Smemo S, Tena JJ, Kim KH, Gamazon ER, Sakabe NJ, Gomez-Marin C, et al Obesity-associated variants within *FTO* form long-range functional connections with *IRX3* . Nature. 2014;507(7492):371–5. 10.1038/nature13138 24646999 PMC4113484

[19] Hubacek JA, Stanek V, Gebauerova M, Pilipcincova A, Dlouha D, Poledne R, et al A *FTO* variant and risk of acute coronary syndrome. Clin Chim Acta. 2010;411(15–16):1069–72. 10.1016/j.cca.2010.03.037 20362563

[20] Lappalainen T, Kolehmainen M, Schwab US, Tolppanen AM, Stancakova A, Lindstrom J, et al Association of the *FTO* gene variant (rs9939609) with cardiovascular disease in men with abnormal glucose metabolism—the Finnish Diabetes Prevention Study. Nutr Metab Cardiovasc Dis. 2011;21(9):691–8. 10.1016/j.numecd.2010.01.006 20400278

[21] Ahmad T, Chasman DI, Mora S, Pare G, Cook NR, Buring JE, et al The *fat-mass and obesity-associated* (*FTO*) gene, physical activity, and risk of incident cardiovascular events in white women. Am Heart J. 2010;160(6):1163–9. 10.1016/j.ahj.2010.08.002 21146673 PMC3058560

[22] Kivimaki M, Smith GD, Timpson NJ, Lawlor DA, Batty GD, Kahonen M, et al Lifetime body mass index and later atherosclerosis risk in young adults: examining causal links using Mendelian randomization in the Cardiovascular Risk in Young Finns study. Eur Heart J. 2008;29(20):2552–60. 10.1093/eurheartj/ehn252 18550552 PMC2567023

[23] Maganti K, Rigolin VH, Sarano ME, Bonow RO. Valvular heart disease: diagnosis and management. Mayo Clin Proc. 2010;85(5):483–500. 10.4065/mcp.2009.0706 20435842 PMC2861980

[24] Owens DS, Katz R, Takasu J, Kronmal R, Budoff MJ, O'Brien KD. Incidence and progression of aortic valve calcium in the Multi-ethnic Study of Atherosclerosis (MESA). Am J Cardiol. 2010;105(5):701–8. 10.1016/j.amjcard.2009.10.071 20185020 PMC2829478

[25] Funkat A, Beckmann A, Lewandowski J, Frie M, Ernst M, Schiller W, et al Cardiac surgery in Germany during 2013: a report on behalf of the German Society for Thoracic and Cardiovascular Surgery. Thorac Cardiovasc Surg. 2014;62(5):380–92. 10.1055/s-0034-1383430 24995534

[26] Makkar RR, Fontana GP, Jilaihawi H, Kapadia S, Pichard AD, Douglas PS, et al Transcatheter Aortic-Valve Replacement for Inoperable Severe Aortic Stenosis. N Eng J Med. 2012;366(18):1696–704. 10.1056/NEJMoa1202277 22443478

[27] Probst V, Le Scouarnec S, Legendre A, Jousseaume V, Jaafar P, Nguyen JM, et al Familial aggregation of calcific aortic valve stenosis in the western part of France. Circulation. 2006;113(6):856–60. 16461814 10.1161/CIRCULATIONAHA.105.569467

[28] Holle R, Happich M, Löwel H, Wichmann HE, Group MKS. KORA—a research platform for population based health research. Gesundheitswesen. 2005;67 Suppl 1:S19–25. 16032513 10.1055/s-2005-858235

[29] Wichmann HE, Gieger C, Illig T, Group MKS. KORA-gen—resource for population genetics, controls and a broad spectrum of disease phenotypes. Gesundheitswesen. 2005;67 Suppl 1:S26–30. 16032514 10.1055/s-2005-858226

[30] Ye S, Dhillon S, Ke X, Collins AR, Day IN. An efficient procedure for genotyping single nucleotide polymorphisms. Nucleic Acids Res. 2001;29(17):E88–8. 11522844 10.1093/nar/29.17.e88 PMC55900

[31] Gomes I, Collins AR, Lonjou C, Thomas NS, Wilkinson J, Watson M, et al Hardy-Weinberg quality control. Ann Hum Genet. 1999;63:535–8. 11246455 10.1017/S0003480099007824

[32] Graffelman J, Camarena JM. Graphical tests for Hardy-Weinberg equilibrium based on the ternary plot. Hum Hered. 2008;65(2):77–84. 17898538 10.1159/000108939

[33] Hosking L, Lumsden S, Lewis K, Yeo A, McCarthy L, Bansal A, et al Detection of genotyping errors by Hardy-Weinberg equilibrium testing. Eur J Hum Genet. 2004;12(5):395–9. 14872201 10.1038/sj.ejhg.5201164

[34] Nielsen DM, Ehm MG, Weir BS. Detecting Marker-Disease Association by Testing for Hardy-Weinberg Disequilibrium at a Marker Locus. Am J Hum Genet. 1999;63:1531–40. 10.1086/302114 PMC1377570 9867708

[35] Salanti G, Amountza G, Ntzani EE, Ioannidis JP. Hardy-Weinberg equilibrium in genetic association studies: an empirical evaluation of reporting, deviations, and power. Eur J Hum Genet. 2005;13(7):840–8. 15827565 10.1038/sj.ejhg.5201410

[36] Wigginton JE, Cutler DJ, Abecasis GR. A note on exact tests of Hardy-Weinberg equilibrium. Am J Hum Genet. 2005;76(5):887–93. 15789306 10.1086/429864 PMC1199378

[37] Lee WC. Searching for Disease-Susceptibility Loci by Testing for Hardy-Weinberg Disequlibrium in a Gene Bank of Affected Individuals. Am J Epidemiol. 2003;158(5):397–400. 12936892 10.1093/aje/kwg150

[38] Hamm CW, Mollmann H, Holzhey D, Beckmann A, Veit C, Figulla HR, et al The German Aortic Valve Registry (GARY): in-hospital outcome. Eur Heart J. 2014;35(24):1588–98. 10.1093/eurheartj/eht381 24022003 PMC4065384

[39] Jacobsson JA, Danielsson P, Svensson V, Klovins J, Gyllensten U, Marcus C, et al Major gender difference in association of *FTO* gene variant among severely obese children with obesity and obesity related phenotypes. Biochem Biophys Res Commun. 2008;368(3):476–82. 10.1016/j.bbrc.2008.01.087 18249188

[40] Shahid A, Rana S, Saeed S, Imran M, Afzal N, Mahmood S. Common variant of *FTO* gene, rs9939609, and obesity in Pakistani females. Biomed Res Int. 2013;2013:324093 10.1155/2013/324093 24102053 PMC3775403

[41] Wang J, Mei H, Chen W, Jiang Y, Sun W, Li F, et al Study of eight GWAS-identified common variants for association with obesity-related indices in Chinese children at puberty. Int J Obes (Lond). 2012;36(4):542–7. 22083549 10.1038/ijo.2011.218

[42] Zhang M, Zhao X, Cheng H, Wang L, Xi B, Shen Y, et al Age- and sex-dependent association between *FTO* rs9939609 and obesity-related traits in Chinese children and adolescents. PLoS One. 2014;9(5):e97545 10.1371/journal.pone.0097545 24827155 PMC4020831

[43] Scott RA, Bailey ME, Moran CN, Wilson RH, Fuku N, Tanaka M, et al *FTO* genotype and adiposity in children: physical activity levels influence the effect of the risk genotype in adolescent males. Eur J Hum Genet. 2010;18(12):1339–43. 10.1038/ejhg.2010.131 20717169 PMC3002848

[44] Doney AS, Dannfald J, Kimber CH, Donnelly LA, Pearson E, Morris AD, et al The *FTO* gene is associated with an atherogenic lipid profile and myocardial infarction in patients with type 2 diabetes: a Genetics of Diabetes Audit and Research Study in Tayside Scotland (Go-DARTS) study. Circ Cardiovasc Genet. 2009;2(3):255–9. 10.1161/CIRCGENETICS.108.822320 20031593 PMC3045745

[45] Allison AC. Genetic control of resistance to human malaria. Curr Opin Immunol. 2009;21(5):499–505. 10.1016/j.coi.2009.04.001 19442502

[46] Dvorakova V, Bartenschlager H, Stratil A, Horak P, Stupka R, Citek J, et al Association between polymorphism in the *FTO* gene and growth and carcass traits in pig crosses. Genet Sel Evol. 2012;44:13 10.1186/1297-9686-44-13 22510482 PMC3369214

[47] Bergman SM, Noble EP. The *D* _*2*_ *Dopamine Receptor* (*DRD2*) Gene and Family Stress; Interactive Effects on Cognitive Functions in Children. Behav Genet. 1997;27(1):33–43. 9145542 10.1023/a:1025611208475

[48] Madrid GA, MacMurray J, Lee JW, Anderson BA, Comings DE. Stress as a mediating factor in the association between the DRD2 *TaqI* polymorphism and alcoholism. Alcohol. 2001;23:117–22. 11331109 10.1016/s0741-8329(00)00138-5

[49] Zhang S-S, Kim K-H, Rosen A, Smyth JW, Sakuma R, Degado-Olguin P, et al *Iroquois homeobox gene 3* establishes fast conduction in the cardiac His-Purkinje network. Proc Natl Acad Sci U S A. 2011;108(33):13576–81. 10.1073/pnas.1106911108 21825130 PMC3158173

[50] Kostin S, Dammer S, Hein S, Klovekorn WP, Bauer EP, Schaper J. Connexin 43 expression and distribution in compensated and decompensated cardiac hypertrophy in patients with aortic stenosis. Cardiovasc Res. 2004;62(2):426–36. 15094362 10.1016/j.cardiores.2003.12.010

[51] Gaborit N, Varro A, Le Bouter S, Szuts V, Escande D, Nattel S, et al Gender-related differences in ion-channel and transporter subunit expression in non-diseased human hearts. J Mol Cell Cardiol. 2010;49(4):639–46. 10.1016/j.yjmcc.2010.06.005 20600101

[52] Falcao-Pires I, Hamdani N, Borbely A, Gavina C, Schalkwijk CG, van der Velden J, et al Diabetes mellitus worsens diastolic left ventricular dysfunction in aortic stenosis through altered myocardial structure and cardiomyocyte stiffness. Circulation. 2011;124(10):1151–9. 10.1161/CIRCULATIONAHA.111.025270 21844073

[53] Kamalesh M, Ng C, El Masry H, Eckert G, Sawada S. Does diabetes accelerate progression of calcific aortic stenosis? Eur J Echocardiogr. 2009;10(6):723–5. 10.1093/ejechocard/jep048 19406839

[54] Natorska J, Wypasek E, Grudzien G, Sobczyk D, Marek G, Filip G, et al Does diabetes accelerate the progression of aortic stenosis through enhanced inflammatory response within aortic valves? Inflammation. 2012;35(3):834–40. 10.1007/s10753-011-9384-7 21935671 PMC3332381

[55] Otto CM, Prendergast B. Aortic-valve stenosis—from patients at risk to severe valve obstruction. N Engl J Med. 2014;371(8):744–56. 10.1056/NEJMra1313875 25140960

