# Supplementary material for: FTO Is Associated with Aortic Valve Stenosis in a Gender Specific Manner of Heterozygote Advantage: A Population-Based Case-Control Study
Source: PLoS One. 2015 Oct 2;10(10):e0139419. doi: 10.1371/journal.pone.0139419 (PMC4592246; doi:10.1371/journal.pone.0139419)
Supplement: S1 Table — (PDF) [file pone.0139419.s001.pdf]

S1 Table. Primer Sequences for Genotyping AVS Cases.

FTO SNP	Allele	Primer	Primer sequence 5' → 3'	PCR product [bp]
rs9939609	A	fwd-inner	TAGGTTCCTTGCGACTGCTGTGAATATA	201
		rev-outer	AGCCTCTCTACCATCTTATGTCCAAACA	
	T	rev-inner	GAGTAACAGAGACTATCCAAGTGCATCTCA	294
		fwd-outer	GGTTCTACAGTTCCAGTCATTTTTGACAG	
rs8050136	A	fwd-inner	GGCATGCCAGTTGCCCACTGTGGCAGTA	155
		rev-outer	GAGGTGCCATTCCTCAATAATTGGCTCTCG	
	C	rev-inner	GGCAAAAACCACAGGCTCAGATACTG	237
		fwd-outer	CAACCAAGGTCCTTATAGGAAGAGCTTGTG	
rs17817449	T	fwd-inner	GTGTTTCAGCTTGGCACACAGAAGCT	221
		rev-outer	CAAAACTGCACTGGTACCCCTTACATTT	
	G	rev-inner	TTAAAGGAGCTGGACTGTTAAATTAAAGCC	148
		fwd-outer	TCCATCTAAGTGCCTTACGGTGAAGAG	

Fwd-inner=forward inner primer; fwd-outer=forward outer primer; rev-inner=reverse inner primer; rev-outer=reverse outer primer.
