# Supplementary material for: FTO Is Associated with Aortic Valve Stenosis in a Gender Specific Manner of Heterozygote Advantage: A Population-Based Case-Control Study
Source: PLoS One. 2015 Oct 2;10(10):e0139419. doi: 10.1371/journal.pone.0139419 (PMC4592246; doi:10.1371/journal.pone.0139419)
Supplement: S2 Table — (PDF) [file pone.0139419.s002.pdf]

S2 Table. Hardy-Weinberg Equilibrium in Genotypic Frequencies of *FTO* rs9939609 in AVS Cases and KORA Controls.

Gender	Genotype distribution AVS cases n [%] KORA controls n [%]			
	TT	TA	AA	*p*-value
All	117 [39.0]	118 [39.3]	65 [21.7]	0.001
(n=729)	135 [31.5]	222 [51.7]	72 [16.8]	0.277
Male	70 [35.9]	74 [37.9]	51 [26.2]	0.001
(n=495)	93 [31.0]	156 [52.0]	51 [17.0]	0.346
Female	47 [44.8]	44 [41.9]	14 [13.3]	0.516
(n=234)	42 [32.6]	66 [51.2]	21 [16.3]	0.717
