# Supplementary material for: FTO Is Associated with Aortic Valve Stenosis in a Gender Specific Manner of Heterozygote Advantage: A Population-Based Case-Control Study
Source: PLoS One. 2015 Oct 2;10(10):e0139419. doi: 10.1371/journal.pone.0139419 (PMC4592246; doi:10.1371/journal.pone.0139419)
Supplement: S3 Table — (PDF) [file pone.0139419.s003.pdf]

S3 Table. *FTO* rs9939609, rs8050136 and rs17817449 Genotype Frequencies in AVS Cases.

		Genotype distribution AVS cases n [%]		
Gender	*FTO* SNP	11[1]	12[1]	22[1]
All (n=300)	rs9939609	117 [39.0]	118 [39.3]	65 [21.7]
	rs8050136	117 [39.0]	118 [39.3]	65 [21.7]
	rs17817449	117 [39.0]	118 [39.3]	65 [21.7]
Male (n=195)	rs9939609	70 [35.9]	74 [37.9]	51 [26.2]
	rs8050136	70 [35.9]	74 [37.9]	51 [26.2]
	rs17817449	70 [35.9]	74 [37.9]	51 [26.2]
Female (n=105)	rs9939609	47 [44.8]	44 [41.9]	14 [13.3]
	rs8050136	47 [44.8]	44 [41.9]	14 [13.3]
	rs17817449	47 [44.8]	44 [41.9]	14 [13.3]

[1]11=low-risk genotype, 12=heterozygote-risk genotype, 22=high-risk genotype.
