# Supplementary material for: FTO Is Associated with Aortic Valve Stenosis in a Gender Specific Manner of Heterozygote Advantage: A Population-Based Case-Control Study
Source: PLoS One. 2015 Oct 2;10(10):e0139419. doi: 10.1371/journal.pone.0139419 (PMC4592246; doi:10.1371/journal.pone.0139419)
Supplement: S4 Table — (PDF) [file pone.0139419.s004.pdf]

S4 Table. Odds Ratios (OR) and 95% Confidence Intervals (CI) of *FTO* rs9939609 Between AVS Cases and KORA Controls.

		All (n=729)				Female (n=234)			
		Unadjusted		Adjusted[1]		Unadjusted		Adjusted[1]	
Genetic model	Genotype	OR	[95% CI]	OR	[95% CI]	OR	[95% CI]	OR	[95% CI]
Co-dominant	TT	1		1		1		1	
	TA	0.614	[0.433-0.868]	0.691	[0.464-1.028]	0.598	[0.326-1.089]	1.145	[0.539-2.482]
	AA	1.042	[0.672-1.614]	1.069	[0.652-1.752]	0.598	[0.247-1.413]	1.110	[0.374-3.273]
Recessive	TT+TA	1		1		1		1	
	AA	1.371	[0.926-2.027]	1.315	[0.844-2.046]	0.792	[0.351-1.740]	1.023	[0.379-2.681]
Overdominant	TT+AA	1		1		1		1	
	TA	0.605	[0.443-0.824]	0.675	[0.472-0.962]	0.162	[0.188-0.201]	1.110	[0.560-2.225]

[1]Values were adjusted for age, BMI, diabetes mellitus and hypertension.
