# Supplementary material for: FTO Is Associated with Aortic Valve Stenosis in a Gender Specific Manner of Heterozygote Advantage: A Population-Based Case-Control Study
Source: PLoS One. 2015 Oct 2;10(10):e0139419. doi: 10.1371/journal.pone.0139419 (PMC4592246; doi:10.1371/journal.pone.0139419)
Supplement: S5 Table — (PDF) [file pone.0139419.s005.pdf]

S5 Table. Odds Ratios (OR) and 95% Confidence Intervals (CI) of *FTO* rs9939609 Between 368 Male AVS Cases and KORA Controls, Age 50-70 Years.

		Unadjusted			Adjusted[1]		
Genetic model	Genotype	OR	[95% CI]	*p*-value	OR	[95% CI]	*p*-value
Co-dominant	TT	1		0.002	1		0.007
	TA	0.452	[0.232-0.870]		0.419	[0.216-0.798]	
	AA	1.351	[0.666-2.723]		1.168	[0.568-2.370]	
Recessive	TT+TA	1		0.002	1		0.062
	AA	2.059	[1.090-3.820]		1.839	[0.958-3.463]	
Overdominant	TT+AA	1		0.001	1		0.002
	TA	0.401	[0.220-0.713]		0.395	[0.216-0.702]	

[1]Values were adjusted for age, BMI, diabetes mellitus and hypertension.
