# Supplementary material for: FTO Is Associated with Aortic Valve Stenosis in a Gender Specific Manner of Heterozygote Advantage: A Population-Based Case-Control Study
Source: PLoS One. 2015 Oct 2;10(10):e0139419. doi: 10.1371/journal.pone.0139419 (PMC4592246; doi:10.1371/journal.pone.0139419)
Supplement: S1 File — (PDF) [file pone.0139419.s006.pdf]

Supporting Information

FTO is Associated with Aortic Valve Stenosis in a Gender Specific Manner of Heterozygote Advantage: A Population-Based Case-Control Study

Cindy Thron[1], Payam Akhyari[2], Erhard Godehardt[2], Artur Lichtenberg[2], Ulrich Rüther[1], Stefanie Seehaus[1*]

[1]Institute for Animal Developmental and Molecular Biology, Heinrich-Heine-University, Düsseldorf, Germany

[2]Department of Cardiovascular Surgery, Heinrich-Heine-University, Düsseldorf, Germany

[*]Corresponding author:

E-mail: Stefanie.Seehaus@hhu.de

SI Methods

Pilot Study

In a pilot study, 71 pilot AVS cases and 442 pilot controls of a previously published control group [1] were analyzed. An association of *FTO* rs1121980 (in linkage with rs9939609) with AVS of heterozygote advantage was apparent applying the co-dominant model. Based on these data the required sample size for a study power of 80% was calculated with $\chi 2$ test by comparing genotype frequencies of AVS pilot cases with pilot controls under specification of number of participants (G*Power, www.gpower.hhu.de). A sample size of 255 AVS cases was calculated.

As recent publications focused on *FTO* rs9939609, we also performed the initial study for rs9939609. No further analysis were performed in the pilot study. Data of the pilot study (pilot AVS cases and pilot controls) have not been used in the current study.

Screening and identification of patients with AVS

All patients admitted to the Department of Cardiovascular Surgery at the University Hospital Düsseldorf for operative therapy of aortic valve disease were screened for inclusion in the current study. All patients included in this study were enrolled in a prospective manner. Correct inclusion of patients was founded on three independent screening columns:

1) All patients underwent a thorough evaluation by transthoracic echocardiography (TTE) with or without additional transesophageal echocardiography (TEE) to ensure the diagnosis of AVS prior to admission to the hospital for cardiac surgery. In addition to the echocardiography and as a routine procedure, all patients over 50 years old underwent left heart catheterization and selective coronary angiography to look for

relevant coronary artery disease. In a prospective manner medical records and charts of patients admitted for cardiac surgery were screened by P.A. and A.L. for diagnosed AVS. Documented history of rheumatic fever as derived from the medical records was used to diagnose rheumatic AVS.

2) As a second independent diagnostic step, all patients undergoing any procedure for any type of valvular heart disease received an intraoperative TEE, which a) confirms the primary pathology, b) rules out any other undetected abnormality, and c) confirms the procedural success at the end of the operation. The intraoperative TEE was performed and evaluated by a senior attending anesthesiologist, primarily involved in the section of cardiac anesthesia and routinely performing TEEs with specific focus on assessment of valve function. Patients with intraoperative TEE finding diverting from the preoperatively described lesions were excluded from this study.

3) As a third column for the diagnosis of AVS, the macroscopic aspect of the aortic valve was evaluated by the senior surgeon. The latter step was furthermore used to diagnose the presence of a bicuspid aortic valve. The assessment of the senior surgeon involved in the operation was used to further confirm the diagnosis of AVS.

Acquisition of clinical variables

KORA data acquisition has been previously described in detail [2, 3]. For the current study, a data request has been submitted to KORA-gen for the variables: residence in Germany, age at survey, sex, body weight, body length, total cholesterol, HDL, LDL, triglycerides, diabetes mellitus, diastolic and systolic blood pressure, peripheral artery disease and smoking status.

For AVS cases, data points were prospectively collected during the entire study period and in the same or similar fashion as described for KORA controls. In brief, the variables: country of birth, age and sex were registered at the time of hospital admission and rely on personal identification documents. Gender was

rechecked with a SRY-PCR (data not shown). The presence of lipid disorders, diabetes mellitus, history of rheumatic fever and rheumatic heart disease, as well as peripheral artery disease were recorded by manual review of the medical records provided at the admission for cardiac surgery. Blood was collected prior to surgery and peripheral blood lipid profile was determined at the Central Institute of Clinical Chemistry and Laboratory Medicine at the University Hospital Düsseldorf, where preoperative medical records did not already indicate the presence of a lipid disorder. The BMI was calculated measuring height and weight of the patient. Diastolic and systolic blood pressure were measured and recorded upon admission for cardiac surgery. In addition, all AVS cases were tested for infection with HIV, previous or active infection with Hepatitis B and Hepatitis C. Finally, the diagnosis of drug addiction is based on documented events in the medical history. All patients were explicitly asked about their use/abuse of chemical substances and pharmacological drugs. The latter information was requested independently at admission to the hospital ward and in the context of anesthesia premedication. Smoking status was documented via a questionnaire.

Genotyping

Genotyping SNPs via tetra-primer ARMS-PCR allows identification of two alleles in one PCR reaction. Primers were designed with an online primer design program (http://cedar.genetics.soton.ac.uk/public_html/primer1.html) and are listed in the S1 Table. PCR reactions were performed in a total volume of 10 µl containing 30 ng genomic DNA, 1x PCR buffer (5x Green GoTaq® Reaction Buffer, #M7911, Promega), 200 µM deoxyribonucleotide triphosphate (dNTP Mix, #11969064001, Roche), 10 pmol of each inner primer, 1 pmol of each outer primer, and 1 U Taq-Polymerase (5 U/µl Taq-Polymerase in-house production, described earlier [4]). PCR programs were run as follows: denaturation for 2 minutes at 95°C, followed by 30 cycles (rs8050136) or 35 cycles (rs9939609 and rs17817449) for 30 seconds at 95°C, annealing of primers for 30 seconds at 58°C (rs9939609) or 60°C (rs8050136 and rs17817449), elongation of 1 minute at 72°C, followed by 2 minutes at 72°C. PCR products were separated on electrophoretic gels. Expected size of PCR products are listed in the S1 Table.

SI References

1. Hinney A, Nguyen TT, Scherag A, Friedel S, Brönner G, Müller TD, et al. Genome wide association (GWA) study for early onset extreme obesity supports the role of fat mass and obesity associated gene (FTO) variants. PLoS One. 2007;2(12):e1361.

2. Holle R, Happich M, Löwel H, Wichmann HE, Group MKS. KORA--a research platform for population based health research. Gesundheitswesen. 2005;67 Suppl 1:S19-25.

3. Wichmann HE, Gieger C, Illig T, Group MKS. KORA-gen--resource for population genetics, controls and a broad spectrum of disease phenotypes. Gesundheitswesen. 2005;67 Suppl 1:S26-30.

4. Pluthero FG. Rapid purification of high-activity *Taq* DNA polymerase. Nucleic acids research. 1993;21(20):4850-1.
